# Inferential Processing in Autistic and Non-Autistic Readers: New Opportunities for Inquiry

**DOI:** 10.1177/17470218261454262

**Published:** 2026-05-18

**Authors:** Philippa L. Howard, Nicholas Martin, Valerie Benson, Simon P. Liversedge

**Affiliations:** 1 School of Education, University of Bristol, England, UK; 2 School of Psychology, Northumbria University, Newcastle upon Tyne, England, UK

**Keywords:** reading, inference, comprehension, autism

## Abstract

This article discusses inferential processing during reading for autistic and non-autistic readers. We demonstrate the criticality of inferential processing for successful text comprehension, alongside evidence that inferential processing is often less efficient for autistic people relative to non-autistic people. We consider the cognitive mechanisms that may underpin inference generation and highlight the RI-Val (Resonance, Integration and Validation) theory as a potential framework that will allow for considerable theoretical development in this area. The RI-Val theory specifies how validation processes during comprehension are tied to attention shifts, which is a significant development in the conceptualisation of discourse processing. This creates a testable account which, if examined using online methods, provides considerable scope for the development of scientific understanding in relation to the inferential and social-communication differences associated with autism.

## Language and Reading

Although communication occurs across a variety of species, language is a unique human characteristic. The psychological processes that occur to produce and comprehend language are extremely complex. Nonetheless, the majority of the world’s population learns to speak and understand speech through simple exposure to their native language, and, under conditions of effective tuition, most also learn to read and write. Reading is critical for successful and effective function in modern society at both a social and a professional level. Moreover, reading and writing are also, now, one of the primary methods through which people communicate (e.g., text, email, social media and the internet more generally). Written text is also a frequently used method by which humans create permanent records of knowledge to allow transfer from generation to generation via reading. Thus, reading is vital for very many aspects of day-to-day human life. Evidence suggests that for people on the autism spectrum, cognitive processing differences, which currently remain to be fully specified, can prevent the achievement of full and seamless comprehension during reading. In this article, the process of written language comprehension, reading, and how this process might differ between autistic and non-autistic people, is considered. Specifically, this article focuses on the processes associated with the formation of inferences that are necessary to attain a rich and fully specified representation of text meaning.

## What Is Autism and How Does It Affect Reading?

Autism spectrum disorder (henceforth referred to as autism) is a lifelong neurodevelopmental condition characterised by differences in social interaction and communication, and restricted and repetitive patterns of behaviour (American Psychiatric Association, 2013). In relation to linguistic processing, autistic people are often documented to experience difficulties with aural (Kwok et al., 2015) and reading comprehension (Brown et al., 2013; Huemer & Mann, 2010). The reading comprehension difficulties associated with autism can, to a degree, be attributed to differences in low-level language skills such as vocabulary, word decoding, and grammar (often referred to as “core” or “structural” language skills e.g., Wilson & Bishop, 2022a) that vary substantially across the autism community (e.g., Brown et al., 2013; C. Norbury & Nation, 2011; Lindgren et al., 2009; McIntyre et al., 2017; Nation et al., 2006), and which are associated with the development of early reading skills. Impairment of these low-level skills may preclude the processes necessary downstream for effective comprehension to occur. What is relatively unique in the case of autism, is that when low-level skills are intact and proficient, as is very often the case for autistic people without co-occurring learning difficulties, comprehension challenges and/or online text processing differences are often still reported (e.g., Au-Yeung et al., 2018; Howard et al., 2017a; Huemer & Mann, 2010; McIntyre et al., 2017), particularly when comprehension relies upon effective inferential processing (e.g., McIntyre et al., 2020; Micai et al., 2017; Sansosti et al., 2013; Wilson & Bishop, 2020), and are related to social-communication skills (Jones et al., 2009; McIntyre et al., 2018; Ricketts et al., 2013). In our view, this indicates that comprehension challenges associated with autism exist beyond low-level, basic, language processes and also arise from differences in aspects of higher-order language processing, that is, processes associated with the online construction of the discourse representation. In particular, we suggest that differences in the nature of inferential processes are a dominant causative factor with respect to comprehension difficulties in autism, and this is a central contention in this article. Below, the information processing mechanisms that might underpin inferential processing and how these may vary between qualitatively different forms of linguistic inference are considered. We advocate that research that examines how autistic people process a variety of inferences may provide a unique window into understanding reading comprehension and broader social-communication differences associated with autism.

## How Do We Read, and What Are the Inferential Processes Underpinning Reading Comprehension

Over multiple decades, experimental psychologists have conducted scientific research to better understand the nature of the cognitive processes that underpin reading (Rayner, 1998, 2009). Such research has provided the foundation for educational policy and practice for the teaching of literacy in the classroom (Castles et al., 2018; Rayner et al., 2001). Reading is an immensely complex psychological process; it itself comprises multiple sub-processes, requiring the simultaneous engagement and coordination of numerous different psychological systems. In brief, to read, a (sighted) individual must control their eyes to make saccades and fixations. Saccades are when the eyes rotate to move the point of foveal vision from one location/word to another (taking approximately 30 ms during reading), and fixations are when the eyes remain relatively still to process visual information (approximately 250 ms in length during reading). These eye movements move the point of fixation from word to word through a text to permit visual encoding of the orthographic form on the page or screen. These encoded representations then activate abstract lexical representations corresponding to words stored in memory that are identified in order that their syntactic, thematic, and semantic characteristics become available. Through syntactic processing, the structural relationships between the words are computed and by combining the lexical semantic characteristics of words with the developing sentence structure, readers can incrementally construct a shallow representation of the meaning of the sentence. To attain even this basic level of understanding, the reader must engage perceptual (oculomotor, visual, attentional) and linguistic (lexical, syntactic, thematic, and semantic) processing systems, as well as directly call upon short- and long-term memory systems. It is critical to appreciate that to fully comprehend text, the reader must very often go beyond such linguistic processing and engage in higher-order processing, such as inferential processing. Inferential processing is a critical and pervasive aspect of written language comprehension. To illustrate this point, let us consider the following short passage and associated questions in Example 1:

Example 1.

The boy watched the old man dig the hole in the road. He rested on his spade. The soil looked dark, wet and heavy. The work was hard. It was obvious to him that when he left school, he did not want this kind of a job.

What did the old man use to dig the hole? Who rested on a spade? Where did the soil come from? Who was a school student? Who did not want this kind of job?

It is likely that these questions were quick and easy to answer.^1^ However, and perhaps surprisingly, none of the information that is required to enable one to answer the questions is explicitly provided in the text. Instead, readers (quite easily) form a coherent representation of the meaning of the text and correctly answer the questions through the formation of inferences; implicit connections that are critical for comprehension yet go beyond the explicit content of the text. The fact that it is a relatively simple task to answer the questions is a clear indication that readers undertake inferential processing as a matter of course during natural reading. In our example, based on real-world knowledge, we know that spades are often instruments associated with digging, and that an act of digging involves the creation of a hole from which (usually) soil is obtained (though if the event took place on a beach, not a road, then sand rather than soil would likely be obtained). Clearly, readers can rapidly deduce that the old man was using a spade to dig the hole in the road and that the soil must have come out of the newly created hole (Bloom et al., 1990; Haviland & Clark, 1974; Just & Carpenter, 1980). In addition, through the formation of appropriate co-reference relations between the different pronouns (“he,” “him”) and their antecedents that may appear earlier or later in the text (“the boy” and “the old man,” e.g., Cowles & Garnham, 2011; Garnham & Oakhill, 1985; Van Gompel & Liversedge, 2003), readers can compute that the boy was a student at school and did not wish to dig holes in the road in his future life. An important point to make is that upon reading the text and answering the questions, inferential processing occurs so rapidly and automatically that it is usually only after reflection that it even becomes apparent to the reader that none of the information required to answer the questions was provided explicitly in the text. All of the answers generated by the reader were inferred by forming co-reference relations and calling upon existing world knowledge.

What should be evident from the above example, is that inferences during reading can take many forms, from word-based referential links (e.g., forming a co-reference link between “He” and “the old man” in Example 1) through to wider inferences made about global themes of a text that are developed over a period of extended reading and that require the use of world knowledge (e.g., inferring an individual’s intent with respect to their future life). Of course, the observation that people form a diverse range of inferences during reading is not new. There has been a considerable body of work that has resulted in the development of taxonomies and hierarchies for the different types of inferences that readers generate. For example, Graesser et al. (1994) distinguished between text-connecting and knowledge-based inferences, with text-connecting inferences being shallow, low-level inferences constructed from the text base and syntactic information (e.g., referential links), and knowledge-based inferences that involve activation of world knowledge stored in long-term memory (e.g., inferring the consequence of an action). Other accounts include inter-sentence vs. gap-filling inferencing (Oakhill & Cain, 1998); automatic vs. strategic inferencing (McKoon & Ratcliff, 1992); text bound implicit information vs. inferred information (Perfetti & Stafura, 2015); and classifications based on whether an inference leads to an accretion or reduction of information, retrieving or generating knowledge, and is an automatic or controlled process (Guthke, 1991; Kintsch, 1993), as well as earlier alternative taxonomies (e.g., Chikalanga, 1992; Clark, 1977; Harris et al., 1978; Magliano & Graesser, 1991). From this work, we can clearly deduce that there is agreement that many different types of inference are computed during reading, and that the cognitive processes necessary for successful inference generation may differ depending upon the linguistic characteristics of the inference in question. For example, the observation that only some inferences involve access to world knowledge. However, despite this understanding, the nature of processing associated with different types of inference remains to be fully reflected in theoretical and computational specifications of cognitive processing during reading, and this is currently limiting our understanding of inferential processing and how this might differ between autistic and non-autistic people.

## Inference and Autism

People on the autism spectrum often experience difficulties forming inferences during language processing (Loukusa & Moilanen, 2009), which contributes significantly to the difficulties in communication and interaction that autistic people experience on a day-to-day basis (Wilson & Bishop, 2020, 2021, 2022b). As discussed above, this is because of the criticality of inference generation to the formation of a fully specified representation of text meaning, and its necessity for understanding a multitude of social interactions (Cain & Oakhill, 1999; Ford & Milosky, 2008). Studies investigating inferential processing during reading tasks typically indicate that autistic adolescents and adults take longer to form inferences (Jolliffe & Baron-Cohen, 1999; Sansosti et al., 2013), make longer fixations upon and more regressions within inferential texts (Micai et al., 2017; Sansosti et al., 2013), have increased right hemisphere activation when reading inferential texts (Mason et al., 2008), and often fail to form correct inferences relative to comparison groups, when probed via inferential comprehension questions (Le Sourn-Bissaoui et al., 2009; McIntyre et al., 2017; Minshew et al., 1995; Tirado & Saldaña 2016) or when asked to select a missing word or sentence from a series of options (C. Norbury & Nation, 2011; Jolliffe & Baron-Cohen, 1999). It is worth noting that very comparable effects are reported in studies that adopt listening comprehension tasks to study inference processing for autistic adults, adolescents, and children (e.g., Bodner et al., 2015; C. F. Norbury & Bishop, 2002; Dennis et al., 2001; Jolliffe & Baron-Cohen, 2000; Loukusa et al., 2007, 2018; McIntyre et al., 2020; Ozonoff & Miller, 1996; Tesink et al., 2009). The types of inferential processing examined in the studies cited above vary but broadly fall within the category of knowledge-based inferences, such as bridging inferences, where a reader must infer a causal connecting event between two sentences or phrases (e.g., when reading *Sally had forgotten her umbrella. Her hair was soaking wet when she arrived at the office* inferring that it has rained and this is what caused Sally’s hair to be wet), mental state inferences, when a reader must infer a character’s perspective (e.g., inferring that Joan was planning to holiday somewhere with a hot climate when reading *Joan was a real sunworshipper. She searched the internet for her next holiday*), and predictive inferences when readers infer and incorporate a forward prediction into their mental representation of the text (e.g., inferring that the wine glass smashed when reading *Ian dropped his wine glass onto the patio*). Based on the studies cited above, it seems reasonable to suggest that autistic people, in comparison to non-autistic people, often differ in respect of both the efficiency and the effectiveness with which they form inferences and answer inferential questions, particularly when such inferences require processing of world knowledge (knowledge-based inferences) and/or require processing concerning another person’s mental state. We form these conclusions with two caveats. First, the degree to which inference generation differs between autistic and non-autistic readers is moderated by an individual’s basic linguistic skills (C. Norbury & Nation, 2011; C. F. Norbury & Bishop, 2002; Lucas & Norbury, 2015) and age (e.g., Bodner et al., 2015; Loukusa et al., 2007; McIntyre et al., 2020), with increased linguistic skills and age being associated with higher inference comprehension accuracy. Second, differences in the efficiency and effectiveness of inferential processing for autistic readers relative to non-autistic readers are not always neatly coupled or mutually inclusive. For instance, Tirado and Saldaña (2016) used a phrase-by-phrase self-paced reading paradigm to examine whether autistic readers generate inferences about a character’s emotion. Autistic and non-autistic readers had inflated reading times for critical regions of a sentence that were incongruent with an inferred emotion, yet were less accurate in responding to comprehension questions that directly probed their understanding of this emotion. In addition, the reading time effect disappeared for autistic, but not non-autistic readers, when the distance in the text between the sentences that implied the emotion and the critical region was increased. Micai et al. (2017) tracked the eye movements of autistic and non-autistic children and adolescents as they read passages of text and answered questions. Half of the questions probed a global inference (e.g., inferring that “little Mico” was a cat based on the wider text context). Comprehension accuracy for inferential questions was comparable between autistic and non-autistic groups, but the eye tracking data revealed that the efficiency with which autistic adolescents formed global inferences was reduced relative to non-autistic peers. This was evidenced by increased gaze durations (time from when a word is first fixated until a saccade is made to another word) on a critical word that informed the inference, and increased regressions (saccades backwards in the text to allow for re-reading) to the target word when reading inferential comprehension questions. These studies demonstrate that in autistic readers, a decoupling between knowledge-based inference efficiency and effectiveness can occur. To be clear, in circumstances when autistic readers accurately generate knowledge-based inferences, processes required to achieve this appear to be more cognitively effortful, relative to non-autistic participants (also see Saldaña & Frith, 2007; Sansosti et al., 2013). Moreover, it is also possible that autistic readers may show evidence of engaging in inference processing yet fail to maintain or integrate this information sufficiently effectively to accurately answer later inferential questions. What is clear from these studies is the importance of using online methods to study how inference processing unfolds in real time. What remains unclear, however, are the mechanisms that might underpin these different patterns of effects for inferential processing efficiency and effectiveness.

Interestingly, in addition to the above studies, there is also quite consistent evidence to suggest that inference generation does not always differ between autistic and comparison groups during reading, particularly when the inference type can be classified as text-based. For instance, autistic adults demonstrate comparable comprehension accuracy for presuppositions (e.g., inferring that James was previously an omnivore when reading “James stopped eating meat”) and under-informative scalar terms (e.g., correctly interpreting “some” to mean “not all” rather than “all”) relative to comparison groups (Pijnacker et al., 2009). Similar findings are typically reported for listening tasks (Chevallier et al., 2010; Schaeken et al., 2018; Su & Su, 2015). Similarly, the time-course over which autistic adults process a range of different counterfactual statements involving inferred facts (e.g., inferring that “I had not drank enough” when reading “If I had drank enough water. . .”), either does not differ from non-autistic adults, or is quicker, suggesting more efficient processing (Black et al., 2018a, 2018b; Ferguson et al., 2019, 2022). Moreover, Howard, et al. (2017b) reported typical and comparable processing difficulty in the eye movement records of autistic adults and a comparison group, when reading texts that evoke a co-reference link between a category noun (e.g., “bird”) and an atypical (e.g., “penguin”) relative to a typical (e.g., “pigeon”) instance of that category (see also Fajardo et al., 2022). It could perhaps be argued that the inferences involved in presupposition, under-informative scalar, counterfactual, and co-reference processing might be categorised as text-based or “low-level” in the taxonomies introduced in the previous section (e.g., Graesser et al., 1994). Thus, these investigations are somewhat different from those where knowledge-based inferences have been investigated, and where researchers more consistently report effectiveness and/or efficiency differences between autistic people and comparison groups. It therefore seems reasonable to propose that autistic readers may not have a universal inferential processing weakness; rather, the nature of the inference, and the consequential linguistic processing demands, particularly in relation to whether an inference must be formed using world knowledge, may be a critical boundary in respect to determining the presence and magnitude of any inferential processing and performance differences associated with autism. This suggestion raises an interesting question as to how the mechanisms that underpin successful inference generation differ for different inference types during natural reading.

## How Are Inferences Generated During Reading?

A number of theories have been developed to explain semantic processing and the formation of a discourse representation (e.g., Current state strategy; Fletcher & Bloom, 1988; Construction-Integration Model, Kintsch, 1988; Kintsch & van Dijk, 1978; Causal Inference Maker; Van den Broek, 1990; The Structure Building framework; Gernsbacher, 1991, 2013; The Event Indexing Model; Zwaan et al., 1995; The Landscape Model; Tzeng et al., 2005; Van den Broek et al., 1999 Bonding and Resolution; Garrod & Terras, 2000), and several of these share a two-stage view that resonance (similarly referred to as activation by some accounts; a process whereby memory traces related to text are activated, as text is encoded) and integration (a process where encoded text and activated memory traces are combined) underpin comprehension and inference generation during reading. A more recent account, the RI-Val theory (R = Resonance, I = Integration, Val = Validation; Cook & O’Brien 2017; O’Brien & Cook, 2016a, 2016b), incorporates a third and separate process of validation, which is linked to attention shifts, and is a significant development in the conceptualisation of discourse processing.

RI-Val theory proposes that incremental interpretation of text, including inference generation, occurs via the asynchronous onset and parallel completion of three processes: Resonance, Integration, and Validation. These processes can broadly be considered to reflect the formation of causal connections as we read, and, similar to many earlier two-stage models, are assumed to occur incrementally as a reader progresses through the text. As above, resonance refers to passive activation of memory traces corresponding to constituents in text as the text is encoded (see Myers & O’Brien, 1998; O’Brien & Myers, 1999; O’Brien et al., 1998). The assumptions of RI-Val align with earlier-memory based accounts of comprehension (e.g., Kintsch, 1988), such that this resonance process is assumed to be “autonomous, unrestricted, and ‘dumb’” (pp. 252 O’Brien & Cook, 2016b), and that during encoding any relevant traces will be activated (or reactivated) from memory. The strength and speed of resonance will vary between traces, depending upon the level of featural overlap between the encoded information and the trace. Once resonance attains a stable state, the traces that resonate the most are transferred to working memory. Recall that taxonomies of inference (e.g., Graesser et al., 1994; McKoon & Ratcliff, 1992; Oakhill & Cain, 1998; Perfetti & Stafura, 2015) directly reference corresponding and different types of memory traces. For example, knowledge-based inferences (e.g., bridging) primarily require world knowledge resonance, whereas text-connecting inferences (e.g., co-reference) primarily involve resonance of candidate antecedents in the representation of the discourse. Therefore, one might assume the memory stores and nature of the traces that resonate differ according to inference type.

Following the transfer of at least two concepts into working memory via resonance (O’Brien & Cook, 2016b), integration is triggered, whereby encoded text and activated traces are combined to form causal links and maximise meaning coherence (and note that ongoing resonance processing is posited to maintain in parallel until completion). These linkages are assumed to be formed based on general conceptual overlap (*c.f.*, Kintsch, 1988). Following the integration of at least one linkage, the third separate process of validation begins, whereby integrated information (e.g., an inference that has been formed based on encoded text integrated with memory traces) is assessed for featural consistency using a passive pattern matching process (Kamas & Reder, 1995; O’Brien & Cook, 2016b) in relation to prior text and world knowledge. It is this specification of validation as a separate, independent process that distinguishes the RI-Val theory from earlier two-stage accounts. As before, whilst validation occurs, resonance and integration continue to run in parallel until completion. For validation to be successful, the RI-Val theory stipulates that a coherence threshold should be surpassed, and this threshold may vary depending upon a range of text characteristics (Guéraud et al., 2018; Sonia & O’Brien, 2021). Then, and only then, can the reader’s attention shift forward in the text to allow for processing of words that have not yet been visually encoded. From our perspective, it is this aspect of the theory, where attention shifts (that are frequently associated with an overt behavioural response, a saccadic eye movement) are directly tied to specific cognitive events, such as the surpassing of a coherence threshold, that represents the most critical development in relation to accounts of natural reading.

In a final processing step, all three RI-Val processes are specified to run to completion, even after an attention shift has occurred, thereby allowing for the possibility that the properties of previously fixated text might produce effects on words downstream in the text (commonly referred to as “spillover effects”; O’Brien & Cook, 2016a, 2016b). Note that this assumption that resonance, integration, and validation run until completion, even if a coherence threshold has been met, is what distinguishes RI-Val from earlier Standards of Coherence accounts (e.g., van den Broek et al., 2011).

One reason that the RI-Val theory is a step change in theorising associated with inferential processing in reading is that the attention shift mechanism linked to coherence threshold transgression during validation processing is, to our knowledge, the first attempt to directly specify how cognitive inferential processes affect, and to some degree, direct the allocation of visual attention during reading. That is, to us, the RI-Val theory is a first attempt to posit a causative mechanistic account of how higher-level comprehension processes, that include inferential processes, directly affect eye movement behaviour in reading. This theoretical linkage between the coherence threshold and strictures on progressive attention shifts (presumably, frequently characterised in respect of saccades and fixations to upcoming text) is vital because it makes the theoretical account testable (Higgins, 2004). Accordingly, through careful experimentation using eye tracking that is known to provide an online index of moment-to-moment visual and cognitive processes (Liversedge & Findlay, 2000), it is possible to evaluate the process of validation (and, potentially, the processes of resonance and integration), which offers potential for significant progression in relation to scientific understanding of discourse processing in respect of standard accounts of reading, as well as how such processing might differ for autistic readers.

Moreover, there are striking parallels between the RI-Val processes described above and the mechanisms that domain-general cognitive theories of autism, which are not specific to reading or language processing, propose to underpin the autism behavioural phenotype. For example, less efficient or spontaneous integrative processing (Complex Information Processing; Minshew & Goldstein, 1995; Weak Central Coherence; Happé & Frith, 2006), less efficient world knowledge processing (Complex Information Processing; Minshew & Goldstein, 1995), less efficient mental state processing (Theory of Mind; Baron-Cohen et al., 1985, 2025), atypical prediction error processing (e.g., High Inflexible Precision of Prediction Errors; Van de Cruys et al., 2014), and less efficient executive functioning, including comprehension monitoring (Hughes & Russell, 1993; Ozonoff et al., 1991), have all been proposed to underpin autistic information processing differences. When considered in respect to the reading comprehension process, each of these characterisations might be expected to influence how autistic people activate, integrate, and evaluate prior textual or world knowledge. What we hope is evident is that these possible differences in the reading process are analogous to the resonance, integration, and validation mechanisms specified within RI-Val theory. When considering the case of autism, the possible mappings between mechanisms proposed by theories of autism and the three RI-Val processes are a key strength above previous two-stage accounts of comprehension. These mappings allow different autism theory predictions and their interrelations to be investigated and interpreted under a single over-arching theoretical framework that specifies how these mechanisms interact. There are, consequently, multiple potential explanations that are not necessarily mutually exclusive as to how inferential processing may differ between autistic and non-autistic people, and this may (or indeed, likely, will) vary depending upon the linguistic characteristic of the inference in question. There is considerable scope here to use RI-Val theory to functionally characterise different types of inferences. In addition, given that the RI-Val theory provides a more testable account of inference formation during reading, there is a greater possibility to examine and interpret these mechanisms and processes, and to investigate any differences that exist between autistic and non-autistic people with more detail and specificity than has been previously possible. Moreover, the examination of inference formation during reading could provide critical insights into the nature of processing differences associated with autism more generally, which may contribute to our understanding of social-communication differences associated with this condition.

Of course, whilst the RI-Val theory provides a descriptive account of inferential processing in reading, it currently offers no detail as to how these processes operate in relation to the many other visual and linguistic processes that occur simultaneously when we read. For instance, we do not know whether RI-Val processes are initiated after lexical and syntactic processing are complete, or whether they occur in parallel, with perhaps an asynchronous onset, to lexical and syntactic processes. Christofalos et al. (2025) recently demonstrated that the removal of inter-word spacing disrupts predictive inference generation, which presumably indicates that the quality of lexical input may constrain the onset of RI-Val processes, a finding that may be consistent with lexical processing being complete prior to RI-Val process onset. In contrast, Rayner et al. (2004; see also Staub et al., 2007; Warren & McConnell, 2007; Warren et al., 2008) reported effects of validation (via eye movements during reading of sentences containing anomalies) in early, first pass eye movement measures that we know can reflect lexical and syntactic processing effects in reading (Staub & Rayner, 2007). These results suggest that the validation process might occur in parallel with lexical and syntactic processing, but perhaps with asynchronous onset. Recall that low-level reading processes are well documented to occur less efficiently and effectively for many autistic people (e.g., McIntyre et al., 2017). Understanding how low-level processes operate with respect to RI-Val processes is therefore critical not only for our understanding of the reading process in general, but also for our understanding of how low-level differences may interact, mediate, or even preclude inferential processing for autistic people, irrespective of any additional differences in the RI-Val process itself. Clearly, the specification of how RI-Val processes operate in relation to the multitude of other processes that occur during each fixation we make when we read is an important area that requires more research.

## How Might Inferential Processes Be Reflected in Oculomotor Behaviour

It is important to note that, other than the basic specification that attentional progression should not occur until the coherence threshold has been crossed, the RI-Val theory stipulates nothing as to the patterns of eye movement behaviour readers may exhibit during inferential processing. Nonetheless, even this basic stipulation significantly constrains those patterns of eye movements that should occur. For example, any difficulty associated with validation processing that occurs prior to the coherence threshold being surpassed must be reflected in patterns of saccades and fixations that occur immediately before the eyes make a (rightward in English) saccade to fixate words beyond the currently fixated word. Thus, validation difficulty should be captured in first pass measures on the word itself (e.g., single fixation duration, gaze duration), as well as measures that reflect processing associated with earlier text (e.g. regression path reading times and first pass regressions from the word). It is in this way that we consider that the RI-Val theory provides a degree of empirical testability in respect of its theoretical claims.

To illustrate how we consider that the RI-Val theory might allow us to more effectively experimentally investigate inferential processing during reading in autistic and control individuals, we provide the following example. Any delay in a reader progressing rightwards may be driven by a general difference in the efficiency of the validation process, or this could perhaps be driven by differences in the efficiency of resonance and/or integration processes, which then constrain the onset (and completion) of validation (recall that these processes have an asynchronous onset). It seems possible that these dependencies could perhaps be disentangled via the careful manipulation of resonance and/or integration demands within a single inference type or the careful examination of RI-Val processes across different inference types. For instance, if a reduced efficiency in validation underpins inferential processing differences in autism, one might predict a later progression rightwards past portions of text where an inference is assumed to occur (e.g., regression path durations), for autistic readers relative to non-autistic readers, to a similar degree across all inference types (e.g., text-based and knowledge-based). In contrast, if differences in the efficiency of inferential processing are underpinned by less efficient world knowledge processing, we might expect that the magnitude or presence of these differences in progressing past critical text would only be evident for knowledge-based, but not text-based inferences. We are therefore optimistic that by measuring eye movements as autistic and non-autistic readers process text that requires inferences to be formed, and interpreting data using the RI-Val framework, significant theoretical advancements and understanding of the cognitive processing differences associated with autism can be achieved.

To examine RI-Val processes, it will therefore be necessary to consider multiple eye movement measures of fixation behaviour that reflect the precise time-course and nature of validation for different linguistic stimuli. Indeed, work in this area for typically developing readers is already in progress (e.g., Cook & Wei, 2019; Cook et al., 2018). For example, Van Moort et al. (2021) reported different patterns of fixation in readers’ eye movements when validation processes were disrupted because text content conflicted with world knowledge or prior text content. World knowledge inaccuracies had a prolonged and substantive disruptive impact upon processing, relative to text content inaccuracies, with increased fixation times and regression rates observed across both target and spillover regions. However, the target and spillover regions in this study consisted of whole sentences, thereby making it difficult to associate specific patterns of inspection with particular words that are directly implicated in validation processing. Clearly, this research is formative, and further work involving more fine-grained analyses is required to establish how validation processes occur over time and across linguistic constituents.

Furthermore, an important theoretical issue that requires direct examination concerns not only the degree to which readers make regressive eye movements to re-inspect text during validation, but also whether readers are conscious of their engagement in such processes. Recall that several of the eye movement measures we have suggested that might capture validation processes reflect regressive eye movements readers make as they read. Regressions, saccadic movements that are made to re-inspect text that has already been read, seem well suited to support validation processes, given that one of the main functions of such oculomotor behaviour is to enable revisits to prior text that may have caused processing difficulty when initially encountered (e.g., see Booth & Weger, 2013; Inhoff et al., 2019; Schotter et al., 2014). Recall also that within the RI-Val framework, validation is assumed to be passive and occurring outside the conscious control of the reader. Note, however, that readers are, to some degree, aware of their regressive and re-reading behaviour (Hyönä & Nurminen, 2006), and that there are questions as to the circumstances and consequences of when readers are or are not consciously aware that they are re-reading text (e.g., Paape & Vasishth, 2022a, 2022b). On the basis of this current evidence, it appears that at least some regressive behaviour may, potentially, be at odds with the RI-Val assumption that validation is passive. For example, it seems quite plausible that a reader making a regression followed by a single fixation and then a saccade to inspect more rightward, new text, as they continue reading the sentence normally may be unlikely to be consciously aware that they engaged in reinspection behaviour (as per RI-Val specifications). However, if a reader makes an extensive regression to allow for a sustained period of re-reading, it seems that the reader would be much more likely to be aware that they were engaging in such behaviour. To us, these situations may represent quite different types of processing, perhaps with respect to when strategic executive processes interrupt passive processes, and future research is required to better understand any such distinction. Cook and O’Brien (2023) have now proposed a “divergence threshold” that occurs after the coherence threshold has been met, whereby validation may switch from being a passive process to a strategic process. The authors are open that they do not attempt to explain the mechanisms that underpin strategic repair during reading, but there is clearly work to be done to identify under what circumstances such a passive-to-strategic shift may occur, and how this is reflected in the eye movement record. Moreover, autistic people are often reported to engage in more re-reading relative to non-autistic comparison groups (e.g., Howard et al., 2017a, 2017b, 2017c) and that using our characterisation above, this is likely to be re-reading behaviour that autistic people are consciously aware of. Questions in respect to what processes different types of regressive behaviours reflect and what might trigger a shift between passive and strategic processing is therefore also pertinent in respect to understanding how autistic people process text.

## Summary

In this article, we have briefly outlined the basic cognitive processes that occur during reading, detailing the importance of inferential processing for the successful comprehension of a text. We have provided a brief review of the literature evidencing that the processes that underlie written language comprehension may differ for autistic readers relative to non-autistic readers, particularly in respect to inferential processing. We have also emphasised the fundamental role that eye movements play in reading, particularly in relation to how a representation of the written text is delivered (across space and time on a fixation-by-fixation basis) to the brain for subsequent processing. Within the broader historical context of accounts of inferential processing, we have focused on a current theory, the RI-Val theory, that we feel is groundbreaking in providing stipulations as to how aspects of inferential processing take place in relation to patterns of fixations and saccades in reading. And in considering this account, we conclude that future experimentation examining inferential processing using RI-Val as a theoretical framework and online methods of processing, such as eye tracking, will directly contribute to more nuanced and mechanistic theories of inference processing during reading. It seems uncontroversial to suggest that via the consideration of both theories of discourse comprehension and cognitive theories of autism, there is potential for significant developments in our scientific understanding of the cognitive processes that underpin inferential processing for autistic and non-autistic people.

## Notes

1. Expected answers: What did the old man use to dig the hole? A spade; Who rested on a spade? The old man; Where did the soil come from? The hole; Who was a school student? The boy; Who did not want this kind of job? The boy.

## References

[bibr1-17470218261454262] American Psychiatric Association. (2013). Diagnostic and statistical manual of mental disorders (5th ed.). American Psychiatric Association.

[bibr2-17470218261454262] Au-Yeung S. K. Kaakinen J. K. Liversedge S. P. Benson V . (2018). Would adults with autism be less likely to bury the survivors?: An eye movement study of anomalous text reading. Quarterly Journal of Experimental Psychology, 71(1), 280–290. 10.1080/17470218.2017.13226 28434395

[bibr3-17470218261454262] Baron-Cohen S. Leslie A. M. Frith U. (1985). Does the autistic child have a “theory of mind”? Cognition, 21(1), 37–46. 10.1016/0010-0277(85)90022-8 2934210

[bibr4-17470218261454262] Baron-Cohen S. Radecki M. A. Allison C. Tsompanidis A. Greenberg D. M. Warrier V. Lai M. C. Lombardo M. V. Shamay-Tsoory S. Uzefovsky F. Saxe R. Tager-Flusberg H. Klin A. Leslie A. M. White S. J. Frith U. (2025). Do autistic people have degrees of disability in theory of mind? The importance of meta-analytic convergence. Psychological Inquiry, 36(4), 262–267. 10.1080/1047840X.2025.2605593

[bibr5-17470218261454262] Black J. Barzy M. Williams D. Ferguson H. (2018a). Intact counterfactual emotion processing in autism spectrum disorder: Evidence from eye-tracking. Autism Research, 12(3), 422–444. 10.1002/aur.2056 30575292

[bibr6-17470218261454262] Black J. Williams D. Ferguson H. J. (2018b). Imagining counterfactual worlds in autism spectrum disorder. Journal of Experimental Psychology: Learning, Memory, and Cognition, 44(9), 1444. 10.1037/xlm0000500 29389196

[bibr7-17470218261454262] Bloom C. P. Fletcher C. R. Van Den Broek P. Reitz L. Shapiro B. P. (1990). An on-line assessment of causal reasoning during comprehension. Memory & Cognition, 18(1), 65–71. 10.3758/BF03202647 2314230

[bibr8-17470218261454262] Bodner K. E. Engelhardt C. R. Minshew N. J. Williams D. L. (2015). Making inferences: Comprehension of physical causality, intentionality, and emotions in discourse by high-functioning older children, adolescents, and adults with autism. Journal of Autism and Developmental Disorders, 45(9), 2721–2733. 10.1007/s10803-015-2436-3 PMC4555006 25821925

[bibr9-17470218261454262] Booth R. W. Weger U. W. (2013). The function of regressions in reading: Backward eye movements allow rereading. Memory & Cognition, 41(1), 82–97. 10.3758/s13421-012-0244-y 22886737

[bibr10-17470218261454262] Brown H. M. Oram-Cardy J. Johnson A. (2013). A meta-analysis of the reading comprehension skills of individuals on the autism spectrum. Journal of Autism and Developmental Disorders, 43(4), 932–955. 10.1007/s10803-012-1638-1 23054199

[bibr11-17470218261454262] Cain K. Oakhill J. V. (1999). Inference making ability and its relation to comprehension failure in young children. Reading and Writing, 11(5/6), 489–503. 10.1023/a:1008084120205

[bibr12-17470218261454262] Castles A. Rastle K. Nation K. (2018). Ending the reading wars: Reading acquisition from novice to expert. Psychological Science in the Public Interest, 19(1), 5–51. 10.1177/1529100618772271 29890888

[bibr13-17470218261454262] Chevallier C. Wilson D. Happé F. Noveck I. (2010). Scalar inferences in autism spectrum disorders. Journal of Autism and Developmental Disorders, 40(9), 1104–1117. 10.1007/s10803-010-0960-8 20143144

[bibr14-17470218261454262] Chikalanga I. (1992). A suggested taxonomy of inferences for the reading teacher. Reading in a Foreign Language, 8(2), 67044. 10.64152/10125/67044

[bibr15-17470218261454262] Christofalos A. L. Arco N. M. Laks M. Sheridan H. (2025). The impact of interword spacing on inference processing during text reading: Evidence from eye movements. Discourse Processes, 62(1), 1–15. 10.1080/0163853X.2024.2413811

[bibr16-17470218261454262] Clark H. H. (1977). Psychology and language. In LaBerge D. Samuels S.J. (Eds.), Basic processes in reading: Perception & Comprehension (p. 43). Erlbaum, 1977.

[bibr17-17470218261454262] Cook A. E. O’Brien E. J. (2017). Fundamentals of inferencing during reading. Language and Linguistics Compass, 11(7), e12246. 10.1111/lnc3.12246

[bibr18-17470218261454262] Cook A. E. O’Brien E. J. (2023). Cognitive processes involved in text comprehension: Walking the fine line between passive and strategic validating processes in reading. In Zufferey S. Gygax P . (Eds.), The Routledge handbook of experimental linguistics (pp. 235–249). Routledge.

[bibr19-17470218261454262] Cook A. E. Walsh E. K. Bills M. A. Kircher J. C. O’Brien E. J. (2018). Validation of semantic illusions independent of anomaly detection: Evidence from eye movements. Quarterly Journal of Experimental Psychology, 71(1), 113–121. 10.1080/17470218.2016.1264432 27882856

[bibr20-17470218261454262] Cook A. E. Wei W. (2019). What can eye movements tell us about higher level comprehension?. Vision, 3(3), 45. 10.3390/vision3030045 PMC6802807 31735846

[bibr21-17470218261454262] Cowles H. W. Garnham A. (2011). Noun-phrase anaphor resolution: Antecedent focus, semantic overlap, and the informational load hypothesis. In Gibson E. Pearlmutter N. J . (Eds.), The processing and acquisition of reference. (pp. 297–322). Massachusetts Institute of Technology.

[bibr22-17470218261454262] Dennis M. Lazenby A. L. Lockyer L. (2001). Inferential language in high-function children with autism. Journal of Autism and Developmental Disorders, 31(1), 47–54. 10.1023/A:1005661613288 11439753

[bibr23-17470218261454262] Fajardo I. Pérez A. I. Ferrer A. Pérez-Fuster P. García-Blanco A. C . (2022). Anaphor processing during reading comprehension in students with autism spectrum disorder. Reading & Writing Quarterly, 38(6), 505–525. 10.1080/10573569.2021.1982430

[bibr24-17470218261454262] Ferguson H. J. Black J. Williams D. (2019). Distinguishing reality from fantasy in adults with autism spectrum disorder: Evidence from eye movements and reading. Journal of Memory and Language, 106, 95–107. 10.1016/j.jml.2019.03.001

[bibr25-17470218261454262] Ferguson H. J. Wimmer L. Black J. Barzy M. Williams D. (2022) Autistic adults are not impaired at maintaining or switching between counterfactual and factual worlds: An ERP study. Journal of Autism and Developmental Disorders, 52, 349–360. 10.1007/s10803-021-04939-4 PMC8732958 33704612

[bibr26-17470218261454262] Fletcher C. R. Bloom C. P. (1988). Causal reasoning in the comprehension of simple narrative texts. Journal of Memory and Language, 27(3), 235–244. 10.1016/0749-596X(88)90052-6

[bibr27-17470218261454262] Ford J. A. Milosky L. M. (2008). Inference generation during discourse and its relation to social competence: An online investigation of abilities of children with and without language impairment. Journal of Speech, Language, and Hearing Research, 51(2), 367–380. 10.1044/1092-4388(2008/027) 18367683

[bibr28-17470218261454262] Garnham A. Oakhill J. (1985). On-line resolution of anaphoric pronouns: effects of inference making and verb semantics. British Journal of Psychology, 76(3), 385–393. 10.1111/j.2044-8295.1985.tb01961.x

[bibr29-17470218261454262] Garrod S. Terras M. (2000). The contribution of lexical and situational knowledge to resolving discourse roles: Bonding and resolution. Journal of Memory and Language, 42(4), 526–544. 10.1006/jmla.1999.2694

[bibr30-17470218261454262] Gernsbacher M. A. (1991). Cognitive processes and mechanisms in language comprehension: The structure building framework. In Psychology of learning and motivation (Vol. 27, pp. 217–263). Academic Press.

[bibr31-17470218261454262] Gernsbacher M. A. (2013). Language comprehension as structure building. Psychology Press.

[bibr32-17470218261454262] Graesser A. C. Singer M. Trabasso T. (1994). Constructing inferences during narrative text comprehension. Psychological Review, 101(3), 371. 10.1037/0033-295X.101.3.371 7938337

[bibr33-17470218261454262] Guéraud S. Walsh E. K. Cook A. E. O’Brien E. J. (2018). Validating information during reading: The effect of recency. Journal of Research in Reading, 41, S85–S101. 10.1111/1467-9817.12244

[bibr34-17470218261454262] Guthke T. (1991). Psychologische Untersuchungen zu Inferenzen beim Satz- and Textverstehen [Psychological studies of inferences in sentence and text comprehension] [Unpublished doctoral dissertation, Humboldt Universität].

[bibr35-17470218261454262] Happé F. Frith U . (2006). The weak coherence account: Detail-focused cognitive style in autism spectrum disorders. Journal of Autism and Developmental Disorders, 36(1), 5–25. 10.1007/s10803-005-0039-0 16450045

[bibr36-17470218261454262] Harris Richard J. Kansas State U Monaco Gregory E. Kansas State U. (1978). Psychology of pragmatic implication: Information processing between the lines. Journal of Experimental Psychology: General, 107(1), 1–22. 10.1037/0096-3445.107.1.1

[bibr37-17470218261454262] Haviland S. E. Clark H. H. (1974). What’s new? Acquiring new information as a process in comprehension. Journal of Verbal Learning and Verbal Behavior, 13(5), 512–521. 10.1016/S0022-5371(74)80003-4

[bibr38-17470218261454262] Higgins E. T. (2004). Making a theory useful: Lessons handed down. Personality and Social Psychology Review, 8(2), 138–145. 10.1207/s15327957pspr0802_7 15223513

[bibr39-17470218261454262] Howard P. L. Liversedge S. P. Benson V. (2017a). Investigating the use of world knowledge during on-line comprehension in adults with autism spectrum disorder. Journal of Autism and Developmental Disorders, 47(7), 2039–2053. 10.1007/s10803-017-3129-x 28432451

[bibr40-17470218261454262] Howard P. L. Liversedge S. P. Benson V. (2017b). Processing of co-reference in autism spectrum disorder. Autism Research, 10(12), 1968–1980. 10.1002/aur.1845 28834350

[bibr41-17470218261454262] Howard P. L. Liversedge S. P. Benson V. (2017c). Benchmark eye movement effects during natural reading in autism spectrum disorder. Journal of Experimental Psychology: Learning, Memory, and Cognition, 43(1), 109. 10.1037/xlm0000289 27504678

[bibr42-17470218261454262] Huemer S. V. Mann V. (2010). A comprehensive profile of decoding and comprehension in autism spectrum disorders. Journal of Autism and Developmental Disorders, 40(4), 485–493. 10.1007/s10803-009-0892-3 PMC2837163 19915968

[bibr43-17470218261454262] Hughes C. Russell J. (1993). Autistic children’s difficulty with mental disengagement from an object: Its implications for theories of autism. Developmental Psychology, 29(3), 498–510.

[bibr44-17470218261454262] Hyönä J. Nurminen A. M. (2006). Do adult readers know how they read? Evidence from eye movement patterns and verbal reports. British Journal of Psychology, 97(1), 31–50. 10.1348/00712605X53678 16464286

[bibr45-17470218261454262] Inhoff A. W. Kim A. Radach R. (2019). Regressions during reading. Vision, 3(3), 35. 10.3390/vision3030035 PMC6802794 31735836

[bibr46-17470218261454262] Jolliffe T. Baron-Cohen S. (1999). A test of central coherence theory: Linguistic processing in high-functioning adults with autism or Asperger syndrome: is local coherence impaired?. Cognition, 71(2), 149–185. 10.1016/S0010-0277(99)00022-0 10444907

[bibr47-17470218261454262] Jolliffe T. Baron-Cohen S. (2000). Linguistic processing in high-functioning adults with autism or Asperger’s syndrome. Is global coherence impaired? Psychological Medicine, 30(5), 1169–1187. 10.1017/S003329179900241X 12027052

[bibr48-17470218261454262] Jones C. R. Happé F. Golden H. Marsden A. J. Tregay J. Simonoff E. Pickles A. Baird G. Charman T. (2009). Reading and arithmetic in adolescents with autism spectrum disorders: peaks and dips in attainment. Neuropsychology, 23(6), 718. 10.1037/a0016360 19899830

[bibr49-17470218261454262] Just M. A. Carpenter P. A. (1980). A theory of reading: From eye fixations to comprehension. Psychological Review, 87(4), 329. 10.1037/0033-295X.87.4.329 7413885

[bibr50-17470218261454262] Kamas E. N. Reder L. M. (1995). The role of familiarity in cognitive processing. In Lorch, Jr. R. F. O’Brien E. J. (Eds.), Sources of coherence in reading (pp. 177–202). Lawrence Erlbaum Associates, Inc.

[bibr51-17470218261454262] Kintsch W. (1988). The role of knowledge in discourse comprehension: A construction-integration model. Psychological Review, 95(2), 163. 10.1037/0033-295x.95.2.163 3375398

[bibr52-17470218261454262] Kintsch W. (1993). Information accretion and reduction in text processing: Inferences. Discourse Processes, 16(1–2), 193–202. 10.1080/01638539309544837

[bibr53-17470218261454262] Kintsch W. Van Dijk T. A. (1978). Toward a model of text comprehension and production. Psychological Review, 85(5), 363. 10.1037/0033-295X.85.5.363

[bibr54-17470218261454262] Kwok E. Y. Brown H. M. Smyth R. E. Cardy J. O. (2015). Meta-analysis of receptive and expressive language skills in autism spectrum disorder. Research in Autism Spectrum Disorders, 9, 202–222. 10.1016/j.rasd.2014.10.008

[bibr55-17470218261454262] Le Sourn-Bissaoui S. Caillies S. Gierski F. Motte J . (2009). Inference processing in adolescents with Asperger syndrome: Relationship with theory of mind abilities. Research in Autism Spectrum Disorders, 3(3), 797–808. 10.1016/j.rasd.2009.03.003

[bibr56-17470218261454262] Lindgren K. A. Folstein S. E. Tomblin J. B. Tager-Flusberg H. (2009). Language and reading abilities of children with autism spectrum disorders and specific language impairment and their first-degree relatives. Autism Research, 2(1), 22–38. 10.1002/aur.63 PMC2806306 19358305

[bibr57-17470218261454262] Liversedge S. P. Findlay J. M. (2000). Saccadic eye movements and cognition. Trends in Cognitive Sciences, 4(1), 6–14. 10.1016/s1364-6613(99)01418-7 10637617

[bibr58-17470218261454262] Loukusa S. Leinonen E. Kuusikko S. Jussila K. Mattila M. L. Ryder N. Moilanen I. (2007). Use of context in pragmatic language comprehension by children with Asperger syndrome or high-functioning autism. Journal of Autism and Developmental Disorders, 37(6), 1049–1059. 10.1007/s10803-006-0247-2 17072751

[bibr59-17470218261454262] Loukusa S. Mäkinen L. Kuusikko-Gauffin S. Ebeling H. Leinonen E. (2018). Assessing social-pragmatic inferencing skills in children with autism spectrum disorder. Journal of Communication Disorders, 73, 91–105. 10.1007/s10803-006-0247-2 29576384

[bibr60-17470218261454262] Loukusa S. Moilanen I. (2009). Pragmatic inference abilities in individuals with Asperger syndrome or high-functioning autism. A review. Research in Autism Spectrum Disorders, 3(4), 890–904. 10.1016/j.rasd.2009.05.002

[bibr61-17470218261454262] Lucas R. Norbury C. F. (2015). Making inferences from text: It’s vocabulary that matters. Journal of Speech, Language, and Hearing Research, 58(4), 1224–1232. 10.1044/2015_JSLHR-L-14-0330 25934949

[bibr62-17470218261454262] Magliano J. P. Graesser A. C. (1991). A three-pronged method for studying inference generation in literary text. Poetics, 20, 193–232. 10.1016/0304-422X(91)90007-C

[bibr63-17470218261454262] Mason R. A. Williams D. L. Kana R. K. Minshew N. Just M. A. (2008). Theory of mind disruption and recruitment of the right hemisphere during narrative comprehension in autism. Neuropsychologia, 46(1), 269–280. 10.1016/j.neuropsychologia.2007.07.018 PMC2259382 17869314

[bibr64-17470218261454262] McIntyre N. S. Grimm R. P. Solari E. J. Zajic M. C. Mundy P. C. (2020). Growth in narrative retelling and inference abilities and relations with reading comprehension in children and adolescents with autism spectrum disorder. Autism & Developmental Language Impairments, 5, 2396941520968028. 10.1177/2396941520968028 PMC9620466 36381551

[bibr65-17470218261454262] McIntyre N. S. Oswald T. M. Solari E. J. Zajic M. C. Lerro L. E. Hughes C. Devine R. T. Mundy P. C. (2018). Social cognition and reading comprehension in children and adolescents with autism spectrum disorders or typical development. Research in Autism Spectrum Disorders, 54, 9–20. 10.1016/j.rasd.2018.06.004

[bibr66-17470218261454262] McIntyre N. S. Solari E. J. Gonzales J. E. Solomon M. Lerro L. E. Novotny S. Oswald T. M. Mundy P. C. (2017). The scope and nature of reading comprehension impairments in school-aged children with higher-functioning autism spectrum disorder. Journal of Autism and Developmental Disorders, 47, 2838–2860. 10.1007/s10803-017-3209-y 28624962

[bibr67-17470218261454262] McKoon G. Ratcliff R. (1992). Inference during reading. Psychological Review, 99(3), 440. 10.1037/0033-295X.99.3.440 1502273

[bibr68-17470218261454262] Micai M. Joseph H. Vulchanova M. Saldaña D. (2017). Strategies of readers with autism when responding to inferential questions: An eye-movement study. Autism Research, 10(5), 888–900. 10.1002/aur.1731 27910232

[bibr69-17470218261454262] Minshew N. J. Goldstein G. Siegel D. J. (1995). Speech and language in high-functioning autistic individuals. Neuropsychology, 9(2), 255. 10.1037/0894-4105.9.2.255

[bibr70-17470218261454262] Myers J. L. O’Brien E. J. (1998). Accessing the discourse representation during reading. Discourse Processes, 26(2–3), 131–157. 10.1080/01638539809545042

[bibr71-17470218261454262] Nation K. Clarke P. Wright B. Williams C . (2006). Patterns of reading ability in children with autism spectrum disorder. Journal of Autism and Developmental Disorders, 36(7), 911–919. 10.1007/s10803-006-0130-1 16897396

[bibr72-17470218261454262] Norbury C. Nation K. (2011). Understanding variability in reading comprehension in adolescents with autism spectrum disorders: Interactions with language status and decoding skill. Scientific Studies of Reading, 15(3), 191–210. 10.1080/10888431003623553

[bibr73-17470218261454262] Norbury C. F. Bishop D. V. (2002). Inferential processing and story recall in children with communication problems: A comparison of specific language impairment, pragmatic language impairment and high-functioning autism. International Journal of Language & Communication Disorders, 37(3), 227–251. 10.1080/13682820210136269 12201976

[bibr74-17470218261454262] Oakhill J. Cain K. (1998). Problems in text comprehension: Current perspectives and recent research. In: Reitsma P. Verhoeven L. (Eds.), Problems and interventions in literacy development. Neuropsychology and cognition (Vol. 15). Springer.

[bibr75-17470218261454262] O’Brien E. J. Cook A. E. (2016a). Coherence threshold and the continuity of processing: The RI-Val model of comprehension. Discourse Processes, 53(5–6), 326–338. 10.1080/0163853X.2015.1123341

[bibr76-17470218261454262] O’Brien E. J. Cook A. E. (2016b). Separating the activation, integration, and validation components of reading. In Psychology of Learning and Motivation (Vol. 65, pp. 249–276). Academic Press.

[bibr77-17470218261454262] O’Brien E. J. Myers J. L. (1999). Text comprehension: A view from the bottom up. In Narrative comprehension, causality, and coherence (pp. 41–60). Routledge.

[bibr78-17470218261454262] O’Brien E. J. Rizzella M. L. Albrecht J. E. Halleran J. G. (1998). Updating a situation model: A memory-based text processing view. Journal of Experimental Psychology: Learning, Memory, and Cognition, 24(5), 1200. 10.1037/0278-7393.24.5.1200 9747530

[bibr79-17470218261454262] Ozonoff S. Miller J. N. (1996). An exploration of right-hemisphere contributions to the pragmatic impairments of autism. Brain and Language, 52(3), 411–434. 10.1006/brln.1996.0022 8653388

[bibr80-17470218261454262] Ozonoff S. Pennington B. F. Rogers S. J. (1991) Executive function deficits in high-functioning autistic individuals: relationship to theory of mind. Journal of Child Psychology and Psychiatry and Allied Disciplines, 32(7), 1081–1085. 10.1111/j.1469-7610.1991.tb00351.x 1787138

[bibr81-17470218261454262] Paape D. Vasishth S. (2022a). Does conscious rereading lead to targeted regressions in garden-path sentences? Data from a novel stop-and-reread paradigm. PsyArXiv preprint.

[bibr82-17470218261454262] Paape D. Vasishth S. (2022b). Is reanalysis selective when regressions are consciously controlled? Glossa Psycholinguistics, 1(1), 41. 10.5070/G601139

[bibr83-17470218261454262] Perfetti C. A. Stafura J. Z. (2015). Comprehending implicit meanings in text without making inferences. In O’Brien E. J. Cook A. E. Lorch, Jr. R. F. (Eds.), Inferences during reading (pp. 1–18). Cambridge University Press.

[bibr84-17470218261454262] Pijnacker J. Hagoort P. Buitelaar J. Teunisse J. P. Geurts B. (2009). Pragmatic inferences in high-functioning adults with autism and Asperger syndrome. Journal of Autism and Developmental Disorders, 39(4), 607–618. 10.1007/s10803-008-0661-8 19052858

[bibr85-17470218261454262] Rayner K. (1998). Eye movements in reading and information processing: 20 years of research. Psychological Bulletin, 124(3), 372. 10.1037/0033-2909.124.3.372 9849112

[bibr86-17470218261454262] Rayner K. (2009). Eye movements and attention in reading, scene perception, and visual search. The Quarterly Journal of Experimental Psychology, 62(8), 1457–1506. 10.1080/17470210902816461 19449261

[bibr87-17470218261454262] Rayner K. Foorman B. R. Perfetti C. A. Pesetsky D. Seidenberg M. S. (2001). How psychological science informs the teaching of reading. Psychological Science in the Public Interest, 2(2), 31–74. 10.1111/1529-1006.00004 11878018

[bibr88-17470218261454262] Rayner K. Warren T. Juhasz B. J. Liversedge S. P. (2004). The effect of plausibility on eye movements in reading. Journal of Experimental Psychology: Learning, Memory, and Cognition, 30(6), 1290. 10.1037/0278-7393.30.6.1290 15521805

[bibr89-17470218261454262] Ricketts J. Jones C. R. Happé F. Charman T. (2013). Reading comprehension in autism spectrum disorders: The role of oral language and social functioning. Journal of Autism and Developmental Disorders, 43(4), 807–816. 10.1007/s10803-012-1619-4 22843036

[bibr90-17470218261454262] Saldaña D. Frith U. (2007). Do readers with autism make bridging inferences from world knowledge?. Journal of Experimental Child Psychology, 96(4), 310–319. 10.1016/j.jecp.2006.11.002 17196973

[bibr91-17470218261454262] Sansosti F. J. Was C. Rawson K. A. Remaklus B. L. (2013). Eye movements during processing of text requiring bridging inferences in adolescents with higher functioning autism spectrum disorders: A preliminary investigation. Research in Autism Spectrum Disorders, 7(12), 1535–1542. 10.1016/j.rasd.2013.09.001

[bibr92-17470218261454262] Schaeken W. Van Haeren M. Bambini V. (2018). The understanding of scalar implicatures in children with autism spectrum disorder: Dichotomized responses to violations of informativeness. Frontiers in Psychology, 9, 1266. 10.3389/fpsyg.2018.01266 PMC6064869 30083124

[bibr93-17470218261454262] Schotter E. R. Tran R. Rayner K. (2014). Don’t believe what you read (only once) comprehension is supported by regressions during reading. Psychological Science, 25(6), 1218–1226. 10.1177/0956797614531148 24747167

[bibr94-17470218261454262] Sonia A. N. O’Brien E. J. (2021). Text-based manipulation of the coherence threshold. Discourse Processes, 58(5–6), 549–568. 10.1080/0163853X.2021.1927596

[bibr95-17470218261454262] Staub A. Rayner K. (2007). Eye movements and on-line comprehension processes. The Oxford Handbook of Psycholinguistics, 327, 342.

[bibr96-17470218261454262] Staub A. Rayner K. Pollatsek A. Hyönä J. Majewski H. (2007). The time course of plausibility effects on eye movements in reading: evidence from noun-noun compounds. Journal of Experimental Psychology: Learning, Memory, and Cognition, 33(6), 1162. 10.1037/0278-7393.33.6.1162 17983320

[bibr97-17470218261454262] Su Y. Su L. Y. (2015). Interpretation of logical words in Mandarin-speaking children with autism spectrum disorders: Uncovering knowledge of semantics and pragmatics. Journal of Autism and Developmental Disorders,45(7), 1938–1950. 10.1007/s10803-014-2350-0 25577515

[bibr98-17470218261454262] Tesink C. M. Buitelaar J. K. Petersson K. M. van der Gaag R. J. Kan C. C. Tendolkar I. Hagoort P. (2009). Neural correlates of pragmatic language comprehension in autism spectrum disorders. Brain, 132(7), 1941–1952. 10.1093/brain/awp103 19423680

[bibr99-17470218261454262] Tirado M. J. Saldaña D. (2016). Readers with autism can produce inferences, but they cannot answer inferential questions. Journal of Autism and Developmental Disorders, 46(3), 1025–1037. 10.1007/s10803-015-2648-6 26547920

[bibr100-17470218261454262] Tzeng Y. Van Den Broek P. Kendeou P. Lee C. (2005). The computational implementation of the landscape model: Modeling inferential processes and memory representations of text comprehension. Behavior Research Methods, 37(2), 277–286. 10.3758/bf03192695 16171200

[bibr101-17470218261454262] Van de Cruys S. Evers K. Van der Hallen R. Van Eylen L. Boets B. De-Wit L. Wagemans J. (2014). Precise minds in uncertain worlds: predictive coding in autism. Psychological Review, 121(4), 649. 10.1037/a0037665 25347312

[bibr102-17470218261454262] Van den Broek P . (1990). Causal inferences and the comprehension of narrative texts. Psychology of Learning and Motivation, 25, 175–196.

[bibr103-17470218261454262] Van den Broek P. Bohn-Gettler C. M. Kendeou P. Carlson S. White M. J . (2011). When a reader meets a text: The role of standards of coherence in reading comprehension. In McCrudden. M. T. Magliano. J. P. Schraw G. (Eds.). Text relevance and learning from text (pp. 123–139). Emerald Publishing Limited.

[bibr104-17470218261454262] Van den Broek P. Young M. Tzeng Y. Linderholm T . (1999). The landscape model of reading: Inferences and the online construction of a memory representation. In Goldman S. R. Graesser A. C. van den Broek P. (Eds.), The construction of mental representations during reading (pp. 71–98). Lawrence Erlbaum Associates.

[bibr105-17470218261454262] Van Gompel R. P. Liversedge S. P . (2003). The influence of morphological information on cataphoric pronoun assignment. Journal of Experimental Psychology: Learning, Memory, and Cognition, 29(1), 128. 10.1037/0278-7393.29.1.128 12549589

[bibr106-17470218261454262] Van Moort M. L. Koornneef A. van den Broek P. W . (2021). Differentiating text-based and knowledge-based validation processes during reading: Evidence from eye movements. Discourse Processes, 58(1), 22–41.

[bibr107-17470218261454262] Warren T. McConnell K. (2007). Investigating effects of selectional restriction violations and plausibility violation severity on eye-movements in reading. Psychonomic Bulletin & Review, 14, 770–775. 10.3758/BF03196835 PMC2596917 17972747

[bibr108-17470218261454262] Warren T. McConnell K. Rayner K. (2008). Effects of context on eye movements when reading about possible and impossible events. Journal of Experimental Psychology: Learning, Memory, and Cognition, 34(4), 1001–1010. 10.1037/0278-7393.34.4.1001 PMC2667829 18605885

[bibr109-17470218261454262] Wilson A. C. Bishop D. V. (2021). “Second guessing yourself all the time about what they really mean. . .”: Cognitive differences between autistic and non-autistic adults in understanding implied meaning. Autism Research, 14(1), 93–101. 10.1002/aur.2345 32686325

[bibr110-17470218261454262] Wilson A. C. Bishop D. V. (2022a). A novel online assessment of pragmatic and core language skills: An attempt to tease apart language domains in children. Journal of Child Language, 49(1), 38–59. doi:10.1017/S0305000920000690 33715658

[bibr111-17470218261454262] Wilson A. C. Bishop D. V. M. (2020). Judging meaning: A domain-level difference between autistic and non-autistic adults. Royal Society Open Science, 7(11), 200845. 10.1098/rsos.200845 PMC7735364 33391789

[bibr112-17470218261454262] Wilson A. C. Bishop D. V. M. (2022b). Stage 2 registered report: investigating a preference for certainty in conversation among autistic adults. PeerJ, 10, e13110. 10.7717/peerj.13110 PMC8919847 35295559

[bibr113-17470218261454262] Zwaan R. A. Langston M. C. Graesser A. C. (1995). The construction of situation models in narrative comprehension: An event-indexing model. Psychological Science, 6(5), 292–297. 10.1111/j.1467-9280.1995.tb00513.x

